# Isolation and Detection of the Emerging Pathogen *Escherichia albertii* in Clinical Stool Samples and the Potential Transmission by Meat Samples in Retail

**DOI:** 10.3390/microorganisms12122408

**Published:** 2024-11-23

**Authors:** Muhammad Zeeshan Zafar, Klara De Rauw, Anne-Marie Van den Abeele, Marie Joossens, Lore Heyvaert, Kurt Houf

**Affiliations:** 1 Department of Veterinary and Biosciences, Faculty of Veterinary Medicine, Ghent University, Salisburylaan 133, 9820 Merelbeke, Belgium; muhammadzeeshan.zafar@ugent.be (M.Z.Z.); lore.heyvaert@ugent.be (L.H.); 2 Laboratory of Microbiology, Sint-Lucas Hospital, Groenebriel 1, 9000 Ghent, Belgium; klara.derauw@azstlucas.be (K.D.R.); anne-marie.vandenabeele@azstlucas.be (A.-M.V.d.A.); 3 Laboratory of Microbiology, Department of Biochemistry and Microbiology, Ghent University, Karel Lodewijk Ledeganckstraat 35, 9000 Ghent, Belgium; marie.joossens@ugent.be

**Keywords:** *Escherichia albertii*, meat, stool, reservoir, isolation, detection

## Abstract

The significance of *Escherichia albertii* as a foodborne pathogen is increasingly acknowledged, but the assessment of its occurrence and transmission remains challenging due to the lack of validation of selective isolation, detection, and identification methods. The aim of the present study was to examine its presence on various meat samples at the retail level in order to assess a potential foodborne transmission and its occurrence in clinical stool samples. First, the evaluation and selection of a selective enrichment broth and isolation medium, combined with an optimized identification by MALDI-TOF MS, as well as a suitable DNA extraction method and a PCR-based detection strategy were developed. After the evaluation of existing isolation strategies and the formulation of an adapted enrichment and isolation medium, 100% isolation specificity was not achieved. An identity confirmation of suspected colonies remains necessary. A total of 292 samples, including 45 beef fillet, 51 minced beef, 50 pork fillet, 30 minced pork, 30 chicken carcass, 51 chicken fillet, and 35 minced chicken samples were examined. Samples were all collected at the retail level, including supermarkets and local butcheries. *Escherichia albertii* was isolated from two chicken fillets (3.9%) and additionally detected in one minced chicken (4.5%) and two other chicken fillet (4.5%) samples by a PCR assay. All beef and pork samples tested negative for its presence, but transmission through these meat types cannot be excluded, as it potentially correlates with the level of fecal contamination that was significantly higher on poultry products. With other hygienic conditions and processing steps applied, the presence of *E. albertii* on food can therefore differ in other parts of the world. *Escherichia albertii* was present in 0.4% of the 2419 clinical stool samples examined. The future development of a chromogenic isolation medium, as well as further extensive epidemiologic approaches and a genomic comparison of human, food, and animal isolates, could enhance the assessment of the emerging pathogen status and its potential as a foodborne hazard.

## 1. Introduction

*Escherichia albertii* is an enteric zoonotic pathogen that exhibits a wide range of phenotypic and genetic variations [1]. Most isolates were initially misidentified as *Hafnia alvei* or *Escherichia coli*, but after extensive characterization, including DNA–DNA hybridization and 16S rDNA sequencing, it was reclassified into a new species [2]. The bacterium is a Gram-negative, rod-shaped, facultative anaerobic microorganism belonging to the genus *Escherichia*, which also includes the species *E. coli*, *E. fergusonii*, *E. marmotae,* and five cryptic clades [3]. The lack of motility and the inability to ferment xylose and lactose and to produce β-D-glucuronidase are common biochemical properties of *E. albertii* that can be used to discriminate from *E. coli* [4].

Clinical syndromes associated with *E. albertii* infections in humans include watery diarrhea, stomach pain, and fever, whereas headache and nausea have occasionally been reported [1,5]. The incubation period of an *E. albertii* infection in humans is short, i.e., 12 to 24 h [6]. A rare case of bacteremia has been documented in an elderly woman with underlying comorbidities [7].

Virulence-associated genes present in most *E. albertii* strains include *eae*-encoded intimin [8] and a cytolethal distending toxin encoded by the *cdtABC* operon [9]. The presence of Shiga toxin 2 (Stx2a or Stx2f), encoded by the *stx* gene [10,11], and a porcine attaching–effacing-associated protein encoded by the *paa* gene have also been reported in some *E. albertii* strains [8,12].

*Escherichia albertii* has been isolated worldwide, and outbreaks have been reported in Japan [5]. In one of these outbreaks, the route of transmission was associated with boxed lunches [13], whereas in another, the affected individuals dined at the same restaurant [14]. However, the infection source remained unidentified in most outbreaks or the cause was often first misidentified as *E. coli* [4,15]. Although the clinical importance of *E. albertii* is increasingly acknowledged [5], important aspects of this pathogen, including its prevalence, transmission, pathogenicity, and antimicrobial resistance remain largely undetermined.

The occurrence of this bacterium has been reported in a variety of animals, including wild birds, pets, bats, penguins, seals, and chickens [16]. More recent studies have reported the presence of *E. albertii* in oysters [17], wild raccoons [18], and fattening pigs [19], indicating that *E. albertii* has a wide host range [20,21]. Information on its occurrence in other livestock, including ruminants, as well as in cold-blooded animals, is currently lacking [16]. The clinical significance in animals is largely unknown, but *E. albertii* has been reported as the probable cause of death in redpoll finches (*Carduelis flammea*). Therefore, pathogenicity in animals cannot be ruled out [21]. However, the isolation of *E. albertii* from feces of apparently healthy birds and mammals indicates that they rather act as a potential reservoir.

The significance of *E. albertii* as a foodborne pathogen is still unknown, but the bacterium has been isolated from a variety of foods, including poultry meat, pork, and mutton [22,23,24], soft cheese from raw cow milk (Damietta cheese) [25], lettuce [26], giblets [23], and from drinking and environmental water [27,28].

To isolate and distinguish *E. albertii* from non-*E. albertii* bacteria, different types of agar media have been developed. Maheux et al. [29] initially proposed *E. albertii* medium (mEA) agar for its isolation from stools. Hinenoya et al. [30] supplemented the carbohydrate source lactose in MacConkey agar with xylose, rhamnose, and melibiose. Most *E. albertii* strains do not ferment these sugars, resulting in colourless colonies on the agar medium. Another developed method included deoxycholate hydrogen sulphide lactose agar supplemented with xylose and rhamnose [31].

To enhance the initially low number of bacteria present in food samples, enrichment in buffered peptone water (BPW) [23] or *E. coli* broth (EC broth) [24] has been applied. Arai et al. [31] compared the efficacy of modified *E. coli* (mEC) broth and mEC broth supplemented with novobiocin (NmEC) and reported NmEC as more efficient. Recently, novobiocin–cefixime–tellurite supplemented with modified Tryptic Soy Broth (NCT-mTSB) was proposed to isolate *E. albertii* from poultry meat samples [32]. However, as each study applied different combinations of enrichment broths and isolation media and only a few studies validated the various culture media and conditions, a comparison of data on the presence of *E. albertii* in food is difficult. Over the years, for the detection and identification of *E. albertii* in different matrices, several PCR assays [17,33,34,35,36,37] have been developed, with primer sets targeting a diversity of specific and less specific genes [16].

To assess the potential of *E. albertii* as a foodborne hazard, its possible transmission by raw meat, and the correlation of its occurrence with the presence of fecal contamination on meat, the first aim of this study was to evaluate an isolation and PCR-based detection strategy for *E. albertii* on meat. Secondly, the presence of *E. albertii* on beef, pork, and poultry meat samples at the retail level was examined. At present, screening for *E. albertii* in human patients is not routinely performed, and data regarding its significance are lacking. Therefore, the third aim of the study was to examine stool samples from ambulatory and hospitalized patients with gastrointestinal symptoms in a neighbouring hospital to assess its occurrence.

## 2. Materials and Methods

### 2.1. Bacterial Strains

*Escherichia albertii* and non-*E. albertii* strains used in the present study were obtained from the National Reference Centre for Shiga toxin-producing *E. coli* (NRC STEC) hosted by the clinical microbiology department of Brussels University Hospital UZ Brussel, Belgium, and the BCCM/LMG Bacteria Collection, Ghent University, Belgium (Table 1). Among these, 5 strains, including the type strain, have been isolated from diarrheic stool samples of children in Bangladesh. These strains were previously examined by a polyphasic phenotypic approach, as well as with 16S rDNA sequencing and DNA–DNA hybridization [2]. Strains obtained from the NRC STEC consisted of 8 *E. albertii* isolated from Belgian human stool samples and 6 strains isolated from Japanese birds. The bird isolates originated from the Department of Infectious Diseases, Division of Microbiology, University of Miyazaki, Japan, and have previously been identified by multi-locus sequence analysis, molecular assays identifying *eae* subtypes, LEE integration sites, *stx* subtypes and *cdtB,* and whole-genome sequencing (WGS) [4,37]. Strains isolated from stools of Belgian patients at the NRC STEC have been identified using phenotypic tests including the determination of biochemical properties (xylose, lactose, and beta-glucuronidase) and motility and PCR assays targeting the *E. albertii clpX*, *lysP*, *mdh*, *eae*, and *cdtB* genes, as previously described by De Rauw et al. [38]. In the present study, the identity of all *E. albertii* reference strains has been confirmed using WGS.

### 2.2. E. albertii Identification by MALDI-TOF MS

As of the current manuscript, the Bruker reference spectral databases, including the BDAL MSP-11897 RUO database and the BDAL-IVD MSP-11758 database for MALDI-TOF MS-based species identification with MALDI Biotyper 4.1, released by Bruker Daltonics, Bremen, Germany, consisted of a single strain of *E. albertii* (DSM 17582^T^). To enhance its specificity, an in-house spectral library was constructed using a set of 19 *E. albertii* strains (Table 1). Standardized sample preparation was performed according to the recommendations of Bruker Daltonics. In brief, bacterial colonies of the 3rd generation culture on TSA were suspended in 300 µL of peptone water to obtain a 2 MacFarland solution, followed by the addition of 900 µL of absolute ethanol to inactivate bacteria. The suspension was centrifuged at 13,000× *g* for 2 min and the supernatant was discarded. The pellets were air-dried and 40 µL of 70% formic acid with an equal volume of acetonitrile were added to the mixture and centrifuged at 13,000× *g* for 2 min. To assess technical reproducibility, each sample was spotted eight times on a target plate and air-dried. One microliter of a matrix solution containing 10 mg/mL α-cyano-4-hydroxycinnamic acid in acetonitrile, deionized water, and trifluoroacetic acid (50:47.5:2.5, v v^−1^) was poured onto all spots. The Bruker Bacterial Test Standard (BTS 155 255343; Bruker Daltonics) was used for the calibration of the instrument and to validate the runs. Each spot was subjected to three measurements by averaging the data from the 240 laser shots. Mass spectra ranging from 2000 to 20,000 Da were acquired using a Micro-flex-LT MALDI-TOF MS device (Bruker Daltonics) equipped with a nitrogen laser (11/4337 nm) operating in linear positive ion detection mode under flexControl software (Version 3.4, Bruker Daltonics). Flat line spectra and spectra with the top differing > 0.05% from the main spectrum were removed, retaining at least 21 usable spectra per strain that were downloaded in MALDI Biotyper software to create the main spectrum profiles (MSPs). 

The reliability of the direct smear method was also evaluated for MALDI-TOF MS sample preparation in comparison to the extraction by the formic acid-based method.

### 2.3. Selection of Isolation Media and Optimal Growth Conditions of E. albertii 

First, an in silico evaluation of the currently applied media based on the phenotypic characteristics reported in the literature was performed [29,30], resulting in an adapted isolation medium excluding unnecessary nutrients such as lactose and the expensive selective components rhamnose and melibiose. For the new isolation agar medium (TS-Albertii Agar), 40 g Tryptone Soya Agar (TSA, Oxoid, CM0131B, Basingstoke, UK) in 1 L of distilled water was supplemented with 1.5 g of bile salt mixture (Sigma-Aldrich, Co., St. Louis, MO, USA), 30 mg of neutral red (Sigma-Aldrich), and 1 mg of crystal violet (Merck, KGaA, Darmstadt, Germany) and autoclaved for 15 min at 121 °C. A D-(+)-xylose (Sigma-Aldrich) solution (10 mg xylose/mL distilled water) was first filter-sterilized and added to the autoclaved agar medium at a final concentration of 1% (*w*/*v*).

To increase initially low numbers and to allow the recovery of potentially stressed or injured *E. albertii* cells in food samples, an enrichment broth (TS-Albertii Broth) was selected containing Tryptone Soya Broth (TSB, Oxoid, CM0129B) as basal medium. For this, 30 g of TSB dehydrated powder was mixed with the selective components, 1.5 g bile salt mixture (Sigma-Aldrich), and 1 mg crystal violet (Merck), then dissolved in 1 L of distilled water and autoclaved for 15 min at 121 °C. The final pH of both media was set at 7.3.

To determine optimal growth conditions in the selected media, four *E. albertii* strains (EH2338, 14/1248, NIAH_Bird_23, and LMG 20976^T^ (Table 1) were individually cultured in TSB at 37 °C under aerobic conditions for 24 h and final concentrations were determined by adding 100 μL aliquots of the different dilutions using the spiral plate method onto TSA plates in duplicate. The estimation of bacterial concentrations was performed by calculating the average of two colony counts; meanwhile, the inoculated TSB media were stored at 7 °C. Subsequently, serial 10-fold dilutions in sterile peptone water were prepared to obtain concentrations up to 10^3^ CFU/mL. A hundred microliters of these dilutions was inoculated into 9.9 mL of both TSB and TS-Albertii Broth and incubated at 37 and 41.5 °C for 24 h under aerobic conditions. Subsequently, 100 μL of each dilution was plated in duplicate onto both TSA and TS-Albertii Agar using the spiral plate method and incubated aerobically at 37 and 41.5 °C for 24 h. The plates were then subjected to bacterial counts (Table S1).

### 2.4. Evaluation of Isolation of E. albertii from Meat

As minced chicken is considered a more diversely bacterial contaminated meat compared to beef and pork [39], validation was performed with four biological replicates of minced chicken spiked with four *E. albertii* strains (EH2338, 14/1248, NIAH_Bird_23, and LMG 20976^T^), and repeated once in minced pork (Table 2). The four strains were grown for 24 h in TSB at 37 °C, and concentrations were determined as described in Section 2.3. Meat samples were purchased at the retail level on several occasions. Each time, a 25 g portion was aseptically placed in sterile plastic bags and, subsequently, for each of the four strains, 1 mL was added directly onto the meat to obtain final inoculation levels ranging from 10^6^ to 10^0^ CFU/g. Control samples of each minced chicken and the minced pork meat were also included and here, 1 mL of sterile peptone water was added. The spiked meat samples were placed in a refrigerator at 7 °C to simulate storage under cooled conditions. After 24 h, spiked meat samples were homogenized with 1:10 (*w*/*w*) TS-Albertii Broth for 2 min at 230 rpm using a peristaltic homogenizer (Stomacher^®^ 400 Circulator machine, Seward, UK). Before incubation of the homogenates, 1 mL (3 × 333 μL) of each homogenate was inoculated onto three TS-Albertii Agar plates using the spread plate method and incubated for 24 h at 41.5 °C under aerobic conditions. After incubation of the homogenates for 24 and 48 h at 41.5 °C under aerobic conditions, 100 μL of each dilution was inoculated onto TS-Albertii Agar plates using the spiral plate method and incubated for 24 h at 41.5 °C under aerobic conditions. The plates were examined for the presence of typical colourless colonies and if present, all were subcultured onto TSA plates and further identified by MALDI-TOF MS. Samples were considered positive by isolation if at least one colony was identified as *E. albertii*. Subsequently, 1 mL aliquots of each pre- and post-enrichment homogenate were stored at −20 °C for further *E. albertii* detection by a PCR assay.

### 2.5. Evaluation of E. albertii Detection in Meat by PCR Assay

First, four commercial DNA extraction kits, commonly used in the literature, namely (1) DNeasy^®^ PowerFood^®^ Microbial Kit (Qiagen, Hilder, Germany); (2) DNeasy^®^ Blood and Tissue Kit (Qiagen, Hilder, Germany); (3) NucleoSpin^®^ Food (Machery-Nagel, Duren, Germany); (4) PrepMan^TM^ Ultra (Applied Biosystems, Foster City, CA, USA), and one in-house genomic DNA extraction method based on alkaline lysis, were evaluated to extract DNA from several post-enrichment minced chicken meat samples spiked with the four *E. albertii* strains (EH2338, 14/1248, NIAH_Bird_23, and LMG 20976^T^, Table 1) at 10-fold bacterial dilutions.

Based on the efficiency depending on the cost, time, robustness, and repeatability of the results, the most efficient kit was then selected and used to determine the detection limit of successful PCR amplification by extracting DNA from pre-enrichment homogenates of spiked minced chicken meat samples at 10-fold bacterial dilutions and was further used for the examination of meat samples at the retail level. The selected kit was also used to determine the limit of detection of PCR amplification of post-enrichment chicken carcasses, chicken fillets, minced chicken, beef fillets, minced beef, pork fillets, and minced pork samples spiked with a single *E. albertii* strain (NIAH_Bird_23) at 10-fold bacterial dilutions (Table S2).

For detection in food and animal fecal samples, various studies have applied PCR assays with different primer pairs based on the *clpX*, *lysP*, *mdh*, *yejH*, *yejK*, *eae*, *EAKF1*_ch4033, and *cdt* genes [17,33,34,35,36,37]. Arai et al. [40] developed a real-time PCR assay with primers based on the *EACBF0500* gene (CDS numbers in strain CB9786) that shared a >99% nucleotide sequence identity in the 55 *E. albertii* genomes tested. However, most of these primer sets and target genes have been reported as non-specific [33,41]. The primer set described by Hinenoya et al. [33], targeting the *Eacdt* gene, EaCDTsp-F2 (5′-GCTTAACTGGATGATTCTTG-3′) and EaCDTsp-R2 (5′-CTATTTCCCATCCAATAGTCT-3′), as well as the primer set proposed by Lindsey et al. [35], EA_F (5′-GTAAATAATGCTGGTCAGACGTTA-3′) and EA_R (5′-AGTGTAGAGTATATTGGCAACTTC-3′) targeting a region of a DNA-binding transcriptional activator of a cysteine biosynthesis gene (*EAKF1*_ch4033 from genome KF1, CP007025), were reported as most specific [16]. In the present study, the specificity of the primer sets of Hinenoya et al. [33], Lindsey et al. [35], and Arai et al. [40] were first evaluated using an in silico PCR approach and analyzing the data on the nucleotide blast tool by the National Center for Biotechnology Information database (NCBI; https://blast.ncbi.nlm.nih.gov/Blast.cgi?PROGRAM=blastn&PAGE_TYPE=BlastSearch&LINK_LOC=blasthome/; accessed on 25 December 2023). To evaluate the specificity in vitro, DNA was extracted from all reference strains of *E. albertii* and other non-*E. albertii* species (Table 1) and subjected to PCR amplification using the selected *E. albertii*-specific primer set. To evaluate the sensitivity and the detection limit, DNA was extracted from the previously stored pre-enrichment homogenates of minced chicken meat spiked with four *E. albertii* strains at 10-fold bacterial dilutions.

### 2.6. Examination of Meat Samples at Retail Level

#### 2.6.1. Sample Collection

A total of 292 samples, including 45 beef fillet, 51 minced beef, 50 pork fillet, 30 minced pork, 30 chicken carcass, 51 chicken fillet, and 35 minced chicken samples were collected between May 2022 and April 2024. Samples were all purchased at the retail level, including supermarkets (n = 147) and local butcheries (n = 145), distributed throughout Flanders, Belgium. Samples from the supermarkets were purchased at least two days before the expiry date. All samples were transported in a cool box to the Laboratory of Microbiology, Ghent University, and subjected to microbiological examination within 24 h.

#### 2.6.2. Determination of Fecal Contamination on Meat Samples

To assess the level of fecal contamination present on the meat samples, the number per gram of the indicator bacterium *E. coli* was determined according to the ISO 16649-2 [42]. For each sample, 25 g was taken aseptically in a sterile stomacher bag, homogenized with 1:10 (*w*/*w*) BPW for 2 min at 230 rpm using a peristaltic homogenizer. Then, 1 mL of each homogenate was mixed with Tryptone Bile X-glucuronide agar (TBX, Oxoid, CM0959, Basingstoke, UK) by the pour plating method in duplicate and incubated at 44 °C for 24 h, aerobically. *E. coli* counts were performed by calculating the average of the two colony counts.

#### 2.6.3. Determination of Presence of *E. albertii* on Meat

To isolate *E. albertii* in all 292 samples collected, 25 g of each meat sample was homogenized with 225 mL of TS-Albertii Broth in a sterile stomacher bag for 2 min at 230 rpm using a peristaltic homogenizer (Stomacher^®^ 400 Circulator machine, Seward, UK). For the detection of contamination levels of 10^1^ CFU/g and above, before incubation of the homogenates, 1 mL (3 × 333 μL) of each TS-Albertii Broth homogenate was inoculated onto three TS-Albertii Agar plates by the spread plate method and incubated aerobically at 41.5 °C for 24 h. Following incubation of the TS-Albertii Broth for 24 h at 41.5 °C under aerobic conditions, 100 μL of each homogenate was inoculated onto a TS-Albertii agar plate and incubated as described in Section 2.6.3. Plates were examined for typical colourless colonies and if present, all were subcultured onto TSA plates for further examination by MALDI-TOF MS.

The detection of *E. albertii* by conventional PCR was additionally performed for 222 of the 292 samples, including 37 beef fillet, 35 minced beef, 42 pork fillet, 19 minced pork, 19 chicken carcass, 44 chicken fillet, and 26 minced chicken samples. For this, after 24 and 48 h of enrichment, 1 mL of each homogenate was stored at −20 °C for DNA extraction using the kit selected in the evaluation test as mentioned in Section 2.5. The DNA templates were subjected to PCR amplification using a Veriti^®^ Thermal Cycler (Applied Biosystems, ThermoFisher, Waltham, MA, USA). Selected primers were obtained from Sigma-Aldrich, St. Louis, MO, USA, and diluted to a concentration of 10 μM. The final reaction mixture (25 μL) contained 2 μL of DNA template, 1x PCR buffer (Qiagen, Germany), 100 μM of each deoxyribonucleoside triphosphate (Invitrogen, Carlsbad, CA, USA), 0.375 μM of each primer, and 1.25 U of *Taq* DNA polymerase (Qiagen, Germany). PCR conditions were as described by Lindsey et al. [35], which included an initial denaturation step at 95 °C for 10 min, followed by 30 cycles of 92 °C for 1 min, 57 °C for 1 min, 72 °C for 30 s, and a final elongation step at 72 °C for 5 min. For each experiment, a 2 μL DNA template extracted from a pure culture of *E. albertii* (LMG 20976^T^) was used as a positive control and 2 μL nuclease-free water was added to the PCR mixture as a negative control. The PCR products (5 μL) were size-separated on a 1% agarose gel (Biozym Scientific GmbH, Hessisch Oldendorf, Germany) in 1× Tris–borate–EDTA (TBE) buffer. Electrophoresis was performed at a constant voltage of 75 V for 45 min, followed by staining with 0.5 µg/mL of ethidium bromide (Merck, KGaA, Darmstadt, Germany). Images were captured on ProXima 2650(T) (Isogen Life Science B.V., Utrecht, The Netherlands).

As mentioned in the literature, a few *E. albertii* strains can ferment xylose [36] and therefore display a different colony appearance. To detect xylose-fermenting *E. albertii* strains, a supplementary step involving washing off bacterial colonies in phosphate-buffered saline (PBS) was also implemented in the later stages of this study. The pre- and post-enrichment homogenates yielding bacterial colonies on agar medium were subjected to plate washing. A total of 84 samples, including 9 beef fillet, 10 minced beef, 8 pork fillet, 10 minced pork, 3 chicken carcass, 31 chicken fillet, and 13 minced chicken samples had colonies on the agar plate before enrichment, and a total of 118 samples including 19 beef fillet, 19 minced beef, 22 pork fillet, 9 minced pork, 3 chicken carcass, 31 chicken fillet, and 15 minced chicken samples were found post-enrichment. For this, 1/4th of bacterial growth was washed off in 200 μL of PBS and subsequently subjected to an *E. albertii*-specific PCR assay using the DNeasy^®^ Blood and Tissue Kit (Qiagen) for DNA extraction. Positive isolates were examined by WGS.

### 2.7. Examination of Clinical Stool Samples

#### 2.7.1. Sample Collection

AZ Sint-Lucas is a general secondary care hospital located in Ghent, Belgium. From 24 April 2023 to 30 April 2024, all stool samples (n = 2419) submitted to the clinical laboratory for the detection of common enteric bacterial pathogens were also screened for the presence of *E. albertii* using a culture–PCR combined approach. The majority of the samples were derived from inpatients (n = 1553, 64.2%), with the highest occurrence in the internal medicine department (n = 610, 39.2%), followed by the pediatric department (n = 353, 22.7%) and the geriatric department (n = 278, 17.9%). The patient population consisted of 54.3% (n = 1314) females and 45.6% (n = 1105) males. The age of the study population ranged from 2 months to 100 years, with a median age of 55 years and a mean age of 46 years.

Compliance with ethical standards: as only aggregated and anonymized data have been used, the Helsinki Declaration (2004, revision 2013) on ethical principles for medical research involving human subjects does not apply here.

#### 2.7.2. Isolation of *E. albertii* in Clinical Samples by Culture 

Upon arrival in the laboratory, unpreserved stool specimens were added to FecalSwab medium (Copan Italia, Brescia, Italy) and inoculated onto TS-Albertii Agar plates using a 10 µL inoculation loop. After overnight incubation in an ambient atmosphere at 35 °C, plates were inspected for the presence of colourless colonies. MALDI-TOF MS identification was performed on these xylose-negative colourless colonies using the direct smear method and the extended in-house spectral *E. albertii* library on a MALDI Biotyper sirius instrument (Bruker, Germany). Subsequently, xylose-positive pink colonies were also examined using MALDI-TOF MS if no xylose-negative colonies were present on the plates that tested positive in the *E. albertii* real-time PCR assay (as described in Section 2.7.3).

#### 2.7.3. Detection of *E. albertii* in Cultured Clinical Samples by PCR

To enhance the sensitivity of the *E. albertii* screening in human fecal samples, a real-time PCR method that could easily be embedded in the routine workflow of the clinical microbiology laboratory was implemented. After overnight incubation of the samples on TS-Albertii Agar plates, plates were inspected for growth and a colony sweep was suspended in 1 mL NaCl 0.9% solution and the suspension was used as the DNA template in the real-time PCR without the use of an additional DNA extraction step. Primer and probe sequences for the real-time PCR targeting the *E. albertii*-specific *EACBF0500* gene, EA-real-time PCR F (5′-GGATCGGTTTTCTCTGAAGC-3′), EA-real-time PCR R (5′-CTGCGGTTGCGCTAAGTC-3′), EA-real-time PCR probe (5′-(FAM) TACGGGGACTAACGTTTTGC (BHQ1)-3′), and the 16S rRNA gene of gammaproteobacterial as an internal control (EA-16S rRNA F (5′-CCTCTTGCCATCGGATGTG-3′), EA-16S rRNA R (5′-GGCTGGTCATCCTCTCAGACC-3′) and EA- 16S rRNA probe (5′-(HEX) GTGGGGTAACGGCTCACCTAGGCGAC (BHQ1)-3′) were used as described by Arai et al. [40]. Real-time PCR was performed in a volume of 25 µL consisting of 12.5 µL Takyon^TM^ No ROX Probe 2× MasterMix dTTP (Eurogentec, Seraing, Belgium), 0.75 µL of each 10 µM EA-real-time PCR primer, 0.75 µL of 5µM EA-real-time PCR probe, 0.4 µL of each 10 µM EA-16S rRNA primer, 0.5 µL of 5 µM EA-16S rRNA probe, and 5 µL DNA template. Real-time PCR cycling conditions were used in Rotor-Gene Q and Rotor-Gene 6000 cyclers (Qiagen), with a Takyon activation step at 95 °C for 3 min, followed by 40 cycles of 95 °C for 10 s and 60 °C for 45 s. A colony suspension of *E. albertii* strain 14/1248 diluted in FecalSwab medium and cultured on TS-Albertii Agar was used as a positive control and nuclease-free water was used as a negative control in each real-time PCR run.

### 2.8. Confirmation of PCR Amplicon by DNA Sequencing and E. albertii Isolates by WGS

PCR-positive food isolates and homogenates were sent to the hospital for additional confirmation using real-time PCR and vice versa. Additionally, all food samples generating an amplicon of the predicted base size were purified with NucleoFast 96 PCR Plate, a 96-well ultrafiltration plate for PCR clean-up (Omega Bio-Tek, 400 Pinnacle Way, Suite 450, Norcross, GA, USA), according to the manufacturer’s instructions. They were sent for Sanger sequencing (Eurofins, Ebersberg, Germany). Quality checks and raw sequence reads were trimmed using BioEdit 7.2 software (https://bioedit.software.informer.com/7.2; accessed on 20 May 2024). Subsequently, to obtain the identification, the sequence analysis was performed by NCBI BLAST in the geneBank database (NCBI; https://www.ncbi.nlm.nih.gov; accessed on 20 May 2024).

All strains isolated from food and stools in the present study were also subjected to WGS to confirm the *E. albertii* species. Genomic DNA was extracted using the Maxwell RSC Cultured Cells DNA kit (AS1620, Promega, Madison, WI, USA) on the Maxwell RSC instrument (AS4500, Promega, USA). Paired-end 2 × 150 bp sequencing was performed using the Illumina Novaseq X plus platform at Novogene (Cambridge, UK). The quality of raw data (PE150) was assessed with FastQC (version 0.11.9), developed by the Babraham Institute. Prior to assembly, reads were trimmed (Phred score > Q30) and filtered (length > 50 bp) with fastp 0.23.2 [43], with the correction option enabled. Assembly was performed using Shovill v1.1.0 [44], with SPAdes genome assembler 3.15.4 [45] at its core, and read error correction was disabled. Contigs shorter than 500 bp were removed from the final assembly. The quality of the final assembly, along with its summary statistics including the number of contigs, N50, L50, and percentage of G + C content, was validated using QUAST v.5.2.0 [46]. Genomes were submitted to the Type (Strain) Genome Server (TYGS) [47] to identify the nearest phylogenomic neighbours and calculate the degree of relatedness toward the nearest-neighbour species.

### 2.9. Statistical Analysis

All statistical analyses were performed with GraphPad Prism 10.1.0. (GraphPad Software, Inc., San Diego, CA, USA). The growth of *E. albertii* in TSB and TS-Albertii Broth at 37 and 41.5 °C was analyzed by an analysis of variance (ANOVA) (ordinary one-way with Dunnett’s multiple comparisons test), whereas for *E. coli* counts, the Kruskal–Wallis test was used, followed by Dunn’s post hoc test.

## 3. Results

### 3.1. Evaluation of E. albertii Identification by MALDI-TOF MS 

Before establishing MSPs of the 19 *E. albertii* strains (Table 1), they were subjected to the original Bruker database, and only five were identified correctly. The others were misidentified as *E. coli* with a log score ≥ 2.3. After the introduction of the MSPs of the 19 *E. albertii* strains to the Bruker reference spectral databases, all were correctly identified with a log score ≥ 2.3. Comparison between the extraction method and the direct smear method showed that the direct smear method decreased the time with 1 h of MALDI-TOF MS identification compared to the formic acid-based extraction method but still correctly identified the 19 *E. albertii* strains with a log score ≥ 2.3.

### 3.2. Selection of Isolation Medium and Optimal Growth Conditions for E. albertii from Meat

The mean (±standard deviation) of colony counts of the four *E. albertii* strains (EH2338, 14/1248, NIAH_Bird_23, and LMG 20976^T^ ) cultivated for 24 h in TSB at 37 °C and inoculated onto TSA plates was 9.1 ± 0.06 log_10_ CFU/mL and was not significantly different when cultivated in TSB at 37 °C and inoculated onto TS-Albertii Agar plates; when cultivated in TSB at 41.5 °C and inoculated onto TSA and TS-Albertii Agar plates, mean values (±standard deviation) of 8.9 ± 0.08, 9.0 ± 0.08, and 8.9 ± 0.10 log_10_ CFU/mL, respectively, were obtained. However, significant differences were observed between cultivation for 24 h in TSB at 37 °C, inoculated onto TSA with a mean (±standard deviation) of 9.1 ± 0.06 log_10_ CFU/mL, and cultivation for 24 h in TS-Albertii Broth at 37 °C and inoculated onto TSA and TS-Albertii Agar plates, as well as cultivation in TS-Albertii Broth at 41.5 °C and inoculated onto TSA and TS-Albertii agar plates, with mean values (±standard deviation) of 8.4 ± 0.09, 8.3 ± 0.10, 7.6 ± 0.14, and 7.8 ± 0.15 log_10_ CFU/mL, respectively, as shown in Supplementary Table S1.

Though the cultivation of pure strain enrichment in TSB at 37 °C was shown to be more optimal, for the isolation of *E. albertii* from meat samples, enrichment in TS-Albertii Broth at 41.5 °C was chosen to inhibit the growth of competitive microbiota present on the meat samples.

### 3.3. Evaluation of Isolation of E. albertii from Meat

**Figure 1:**
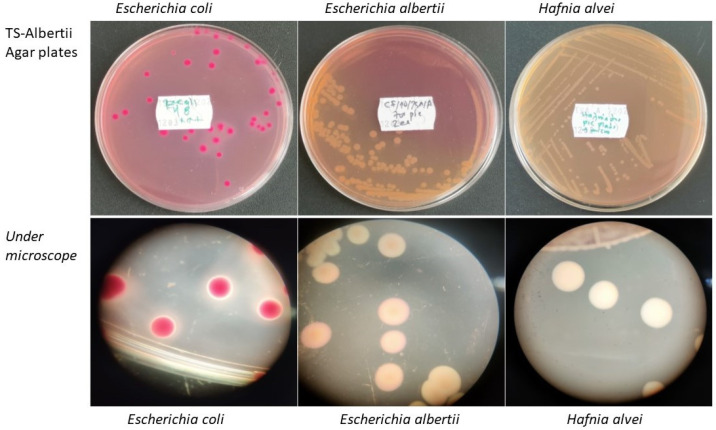
Appearance of colony morphology of *E. coli*, *E. albertii*, and *Hafnia alvei* on TSA-Albertii Agar plates and under light microscope, after isolation, and identified by MALDI-TOF MS.

The detection limit of the isolation method was determined in four biological replicates of minced chicken spiked with four *E. albertii* strains and one in minced pork spiked with a single *E. albertii* strain (NIAH_Bird_23), before and after 24 and 48 h of enrichment in TS-Albertii Broth (Table 2). Before enrichment, all minced chicken and pork meat samples spiked with 10^6^–10^3^ CFU/g meat and directly plated on TS-Albertii Agar yielded presumptive *E. albertii* colourless colonies, which were further confirmed by MALDI-TOF MS. After 24 h enrichment in TS-Albertii Broth, all minced chicken and minced pork meat spiked with less than 10 CFU/g meat yielded colourless colonies on TS-Albertii Agar plates. No difference in the detection limit was observed between the four minced chicken samples spiked separately with the four *E. albertii* strains. However, after 48 h of enrichment, two out of four minced chicken meat samples yielded colourless colonies, which were identified as either *Morganella morganii* or/and *Proteus mirabilis* by MALDI-TOF MS. No colourless colonies from homogenates spiked with sterile peptone water were identified as *E. albertii*. All red colonies on TS-Albertii Agar inoculated before and after enrichment were identified as *E. coli* (Figure 1).

Therefore, an enrichment for 24 h is proposed for the further isolation of *E. albertii* from meat samples, with a detection limit of less than 10 CFU/g, as a longer incubation time of 48 h allowed the overgrowth of background competitive microbiota. 

### 3.4. E. albertii Detection by PCR Assay

Upon blasting the sequences of the primer sets using the nucleotide blast tool by the NCBI, the primer set described by Hinenoya et al. [33], targeting the *Eacdt* gene, revealed non-specific annealing with nucleotide sequences of *E. coli* and *Shigella boydii* with 100% query coverage and identity. The second closest alignment with a 95% query coverage and 100% identity was obtained for *Providencia* spp. and *Neobacillus* spp. The primer set described by Lindsey et al. [35], targeting a region of a DNA-binding transcriptional activator of a cysteine biosynthesis gene (*EAKF1*_ch4033 from genome KF1, CP007025), showed 100% specificity and was therefore selected for further analysis in a conventional PCR assay. The primer set described by Arai et al. [40] also showed 100% specificity by an in silico PCR approach.

The in vitro specificity of the primers was tested by PCR performed using DNA templates of all reference strains (Table 1), and successful PCR amplification (i.e., bands exhibiting amplicons of intended base size) was only obtained for the *E. albertii* strains.

To evaluate the effectiveness of the five DNA extraction methods, DNA was extracted in triplicate from each previously stored post-enrichment homogenate and subjected to an *E. albertii*-specific PCR assay. All were equally effective for extracting DNA from enriched minced chicken samples initially spiked with 10 CFU/g meat, resulting in successful PCR amplification with all four *E. albertii* strains. However, testing before enrichment, the detection limit in minced chicken meat using the DNeasy^®^ Blood and Tissue Kit (Qiagen) was shown to be as high as 10^3^ CFU/g meat for the four *E. albertii* strains, which was consistently lower and with less variable results compared to the other four methods. Its performance was subsequently confirmed on seven different types of meat samples spiked with less than 10 CFU/g meat of a single *E. albertii* strain (NIAH_Bird_23), yielding successful PCR amplification 24 h post-enrichment (Supplementary Table S2). Therefore, DNA extraction using the DNeasy^®^ Blood and Tissue Kit (Qiagen) was applied for further analysis.

### 3.5. Examination of Meat Samples

#### 3.5.1. Determination of Fecal Contamination on Meat Samples

The data for *E. coli* counts were not normally distributed. Therefore, a non-parametric test (Kruskal–Wallis) was used, followed by Dunn’s post hoc test. The *E. coli* counts of the chicken carcasses (2.7 ± 0.64 log_10_ CFU/g, mean ± SD), chicken fillets (1.6 ± 0.55 log_10_ CFU/g), and minced chicken meat (2.0 ± 0.45 log_10_ CFU/g), with maximum ranges of 4.2 log_10_ CFU/g, 2.9 log_10_ CFU/g, and 3.1 log_10_ CFU/g and with minimum ranges of 1.7 log_10_ CFU/g, 1.0 log_10_ CFU/g, and 1.0 log_10_ CFU/g, respectively, were high and significantly different from *E. coli* counts of beef fillets (0.1 ± 0.31 log_10_ CFU/g), minced beef meat (0.2 ± 0.40 log_10_ CFU/g), pork fillets (0.1 ± 0.28 log_10_ CFU/g), and minced pork meat (0.3 ± 0.45 log_10_ CFU/g), with maximum ranges of 1.5 log_10_ CFU/g, 1.2 log_10_ CFU/g, 1.4 log_10_ CFU/g, and 1.4 log_10_ CFU/g and with a minimum range of <10 CFU/g, respectively.

#### 3.5.2. Determination of Presence of *E. albertii* on Meat

Out of 292 samples, *E. albertii* was isolated from two different chicken fillet samples, one from a pre-enrichment homogenate and one from a post-enrichment homogenate (Table 3). The typical colourless colonies on TS-Albertii Agar media, identified as *E. albertii* by MALDI-TOF MS, were also confirmed by WGS. *E. albertii* was not isolated from other meat samples. As shown in Figure 1, red colonies yielded on TS-Albertii Agar were identified as *E. coli,* whereas colourless colonies could be *E. albertii* or *Hafnia alvei*, for which further confirmation by MALDI-TOF MS is required.

As no *E. albertii* was initially isolated, the detection of *E. albertii* by PCR was additionally performed. In this case, one minced chicken sample and two chicken fillet samples, other than those that had a positive isolation, yielded successful PCR amplification for the homogenates after 24 and 48 h of enrichment and were confirmed as *E. albertii*. None of the other meat samples yielded a PCR amplification.

Detection by PCR in plate-washing extracts was additionally performed on 84 samples that had bacterial growth after incubation without enrichment and 118 post-enrichment isolation plates. One chicken fillet sample, also positive by isolation, was PCR-positive by this approach, and all other samples were detected to be negative for *E. albertii*. The PCR-positive food isolates, homogenates, and extracts were also subjected to the real-time PCR assay and tested positive as well.

### 3.6. Examination of Clinical Stool Samples

The clinical stool samples were screened for the presence of *E. albertii* using a real-time PCR on colony suspensions. A total of 10 samples (0.41%) showed *E. albertii*-positive PCR results, out of which seven (0.29%) *E. albertii* colonies were isolated and correctly identified with MALDI-TOF MS. All strains were xylose-negative, and the species identity was confirmed by WGS analysis.

The three samples from which no strain could be isolated had a real-time PCR Ct value higher than 30, indicating a low number of *E. albertii* presence in the culture. Positive PCR results were obtained in April (n = 1), May (n = 1), June (n = 2), July (n = 1), August (n = 2), September (n = 1), and December 2023 (n = 1), as well as in January 2024 (n = 1).

## 4. Discussion

Unlike established foodborne pathogens such as *Campylobacter*, *Salmonella, Listeria*, and STEC, studies on the clinical relevance and epidemiology of *E. albertii* are still scarce. Although the significance of *E. albertii* as an emerging foodborne pathogen is increasingly acknowledged, only a few studies have examined its presence on food and in humans and veterinary clinical samples [2,19,48].

Almost every study has applied different combinations of enrichment broths and isolation media, some without proper validation, hampering the comparison of data and their use in future risk assessment studies. Therefore, in the present study, the effects of temperature, enrichment broth, and isolation agar medium on the growth of *E. albertii* strains in the absence of meat were first evaluated.

The initial methods for isolating *E. albertii* were based on the absence of lactose fermentation. However, Maheux et al. [49] reported that most *E. albertii* strains could ferment lactose, indicating that their prevalence was highly underestimated. Maheux et al. [29] developed an isolation medium for lactose-fermenting and non-fermenting *E. albertii*; however, it was not specific for differentiating between *E. albertii* and certain *E. coli* strains. Hinenoya et al. [30] developed a modified MacConkey agar supplemented with xylose, rhamnose, and melibiose. Based on the inability of *E. albertii* to utilize these sugars compared with *E. coli*, this medium differentiated *E. albertii*. Similarly, different broths, including mEC and N-mEC [31], NCT-mTSB [32], CT-mEC [50], and CTD-TSB [51], have been proposed for the enrichment of *E. albertii*. Moreover, the addition of various combinations of antimicrobial agents have been used as selective substances, but these studies were performed on a limited number of strains and their inhibitory effects were tested under controlled laboratory conditions [31,32,50,51]. As *E. albertii* is not only considered to be present in low numbers but also potentially under stressed conditions induced by low process and storage temperatures, the inclusion of antimicrobial agents in isolation media may hamper its isolation. Based on the biochemical properties of *E. albertii* mentioned in the literature, an adopted isolation medium was developed in the present study, excluding unnecessary and expensive components such as lactose, cellobiose, melibiose, and rhamnose and supplementing general growth media TSA with xylose, bile salt mixture, neutral red, and crystal violet. The bile salt mixture inhibits the growth of non-enteric bacteria, crystal violet has antifungal and antibacterial activity, particularly against Gram-positive bacteria [52], and neutral red is a pH indicator. Most *E. albertii* strains cannot ferment xylose, as they utilize peptones resulting in ammonia production, which raises the pH of the agar medium and therefore, bacterial colonies appear colourless on the agar medium. Besides a selective agar medium for direct isolation, to allow for the isolation of initially low numbers and/or the recovery of stressed *E. albertii* cells present on food, a general enrichment broth (TSB) was supplemented with a bile salt mixture and crystal violet. These two components inhibit the growth of non-enteric bacteria, thereby facilitating the recovery of Gram-negative bacteria.

A non-significant difference in growth performance was observed between the two incubation temperatures. This finding was similar to a study conducted by Arai et al. [31], in which they compared the growth of *E. albertii* at 37 and 42 °C in mEC and NmEC and also observed a non-significant difference in bacterial growth. Similarly, Wakabayashi et al. [32] reported an optimal growth of *E. albertii* strains at 40 °C but reported no significant difference in bacterial growth at temperatures ranging from 37 to 44 °C upon enrichment in NCT-mTSB. Complete inhibition was observed at 46 °C. Therefore, in the present study, incubation at 41.5 °C was applied, as this has an inhibiting effect on the psychro- to mesophilic bacteria present on meat, enhancing the selectivity of the procedure. A significant decrease in the number of cells/mL after incubation was observed at both incubation temperatures when cultured in TS-Albertii Broth compared to a general growth medium (TSB). As the difference between the selective broth and agar medium is just the presence of xylose, this points toward an inhibition due to the general selective substances included, namely bile salts and crystal violet. However, both are commonly included in selective media for *Enterobacteriaceae*, and the inhibition had no significant impact on the detection limit required for the isolation of *E. albertii* from meat samples.

The detection limit of the adapted isolation media was determined by artificially adding 10-fold dilutions of *E. albertii* strains to minced chicken and minced pork meat. Results showed that the detection limit of TS-Albertii Agar was 10^3^ CFU/g for both minced chicken and pork meat without enrichment. This detection limit is lower than that of XRM-MacConkey agar (10^5^ CFU/g), developed by Hinenoya et al. [30] for isolating *E. albertii* from stool samples.

Applying enrichment, the detection limit of the adapted isolation method was less than 10 CFU/g meat. Although the microbial population in chicken meat is higher and more diverse as compared to pork meat [53], the detection limit was the same for minced chicken and pork meat. These results are in agreement with a recent study conducted by Awasthi et al. [54], who reported a detection limit of 4.0 × 10^3^ CFU/mL and 4 CFU/mL before and after enrichment, respectively, for artificially spiked stool samples. Similarly, Wakabayashi et al. [32] reported that the enrichment of meat samples in NCT-mTSB enabled the isolation of *E. albertii* when 1 CFU/g of meat was present. In the present study, the enrichment of artificially spiked chicken meat samples for 48 h showed that an incubation time of more than 24 h allowed for a competitive microbiota overgrowth of *E. albertii*. In this evaluation, the colourless colonies were identified as either *Proteus mirabilis* or *Morganella morganii* by MALDI-TOF MS, indicating that the adapted isolation method is not 100% specific and a further confirmation of colourless colonies is needed.

Concerning identification, initially, MALDI-TOF Bruker reference spectral libraries contained a single *E. albertii* strain (DSM 17582^T^) and failed to correctly identify the *E. albertii* reference strains used in the present study. The inclusion of additional spectra from 19 *E. albertii* strains from different origins resulted in a correct identification of the food and clinical isolates obtained. A comparison between sample preparation by the extraction method and direct smear showed no significant difference in correct identification and log score, which was >2.3, even for the direct smear method, and the latter showed to be more time-efficient.

The specificity of the primer sets proposed by Hinenoya et al. [33] and Lindsey et al. [35] was determined using an in silico PCR approach. The primer set described by Lindsey et al. [35] showed 100% specificity, whereas the *Eacdt* gene-based primers aligned with nucleotide sequences of *E. coli* and *Shigella boydii* with 100% query coverage and identity. However, Hinenoya et al. [33] reported that based on multi-locus sequence analysis, those *Eacdt* gene-positive *S. boydii* strains belonged to a distinct lineage of *E. albertii*. Similarly, the more recent primers designed by Awasthi et al. [54], also based on the *Eacdt* gene, showed alignment with the nucleotide sequences of *E. coli* and *S. boydii* upon blasting using the NCBI tool. Therefore, the primer set described by Lindsey et al. [35] was applied in the present study.

DNA template preparation is crucial for successful PCR amplification, particularly for food samples. Food constituents such as lipids, polysaccharides, enzymes, preservatives, and additives may affect the sensitivity of the PCR assay [55]. The current study evaluated four commercially available kits and an in-house genomic DNA extraction method based on alkaline lysis. PrepMan^TM^ Ultra and the alkaline lysis extraction method are based on heat treatment, whereas the other three included silica-based protocols. Though all five methods performed equally for a successful PCR amplification of the DNA templates extracted from artificially spiked post-enrichment homogenates of minced chicken meat samples spiked with as low of a dose as <10 CFU/g, variable results were obtained for the pre-enriched homogenates. The most inconsistent results were obtained by the alkaline lysis method and the most consistent results at the lowest concentration (10^3^ CFU/g) were obtained with the DNeasy^®^ Blood and Tissue Kit (Qiagen). When only PCR-based detection after enrichment is intended, the alkaline lysis-based method and PrepMan^TM^ Ultra kit showed to be the most time-efficient, whereas the Food kit (Qiagen) was the least time-efficient. Therefore, in the present study, the DNeasy^®^ Blood and Tissue Kit (Qiagen) was selected, as it performed well for all types of meat samples.

In this study, the isolation and detection of *E. albertii* from chicken meat samples, in particular, is in correspondence with previous studies conducted by Asoshima et al. [22] and Hinenoya et al. [48], endorsing the statement that poultry may act as a potential reservoir. Moreover, *E. albertii* was not isolated and detected in beef and pork meat samples, and to our knowledge, no study has reported its presence in pork and beef meat so far. However, Barmettler et al. [19] reported the presence of *E. albertii* from fecal samples of fattening swine; therefore, these food samples cannot totally be excluded as potential infection sources for humans. One explanation is that poultry meat is potentially more fecally contaminated during processing than beef and pork, a statement also supported by the significantly higher number of *E. coli* counts in poultry meat compared to beef and pork.

The inclusion of a plate-washing detection method, by which the bacterial growth on the agar plate from the first serial dilution examined by PCR was observed, showed no added value, as the only successful detection with this method was for a sample from which *E. albertii* was also isolated.

Concerning its clinical relevance and importance as a foodborne pathogen, it is clear that currently, *E. albertii* is a neglected potential pathogen and is still often misidentified as *E. coli* or *Hafnia alvei*. In the present study, the screening of clinical stool samples confirmed its presence in 0.41% of the samples. These samples were also analyzed for the presence of the established enteropathogenic bacteria and viruses by aerobic culture and PCR. In only one of the *E. albertii*-positive samples, an additional pathogen, a sapovirus, was detected. Compared to a previous study conducted by the same hospital on 6774 fecal samples, the occurrence of *E. albertii* ranked lower than the occurrence of *Campylobacter* spp. (5.6%), *Salmonella* spp. (2.0%), and toxigenic *Clostridium difficile* (1.6%), though higher than *Aeromonas* spp. (0.24%), *Yersinia enterocolitica* (0.19%), *Shigella* spp. (0.13%), and *Plesiomonas* spp. (0.05%) [56].

## 5. Conclusions

The present study assessed the occurrence of *E. albertii* in human stool samples and examined its potential transmission by raw meat samples. Even after a validation of existing isolation strategies and an adaptation of an enrichment and isolation medium by eliminating unnecessary and expensive components, 100% specificity was not achieved. An identity confirmation of suspected colonies remains necessary, which is not totally suppressing, as differential colonies on the isolation plates showed to be taxonomic closely related bacteria. The future development of a chromogenic medium would facilitate *E. albertii* research in clinical, veterinary, and food microbiology. The occurrence of *E. albertii* in clinical stools and on retail meat samples in Belgium is low, although transmission through poultry should be taken into account. As its presence correlates with the level of hygiene applied during slaughter and processing, the occurrence of *E. albertii* may be higher in regions with less strict meat hygiene conditions. Also, transmission by other animal reservoirs, including contact with birds, remains a possibility. A further extensive epidemiologic approach including more clinical and food laboratories and the collection and analysis of clinical and general patient parameters, such as food habits and contact with animals, as well as a genomic comparison of human, food, and animal isolates, will contribute to the further assessment of the emerging status of this bacterium.

## Figures and Tables

**Table 1:** Bacterial collection and reference strains used in this study.

Genus	Species	Number of Strains	Strain Origin	Country	Strain Designation
*Escherichia*	*albertii*	5	Human feces	Bangladesh	LMG: 20972-20975, LMG: 20976^T^
	*albertii*	8	Human feces	Belgium	EH2338, EH2349, EH2581, EH2582, EH2675, EH3051, 14/1207, 14/1248
	*albertii*	6	Bird feces	Japan	NIAH_Bird_3, NIAH_Bird_5, NIAH_Bird_8, NIAH_Bird_13 NIAH_Bird_16, NIAH_Bird_23
	*coli*	1	N.A.	N.A.	LMG 33204
	*hermannii*	1	Human toe	United States	LMG 7867^T^
	*vulneris*	1	Rice	Philippines	LMG 20123
	*fergusonii*	1	Human feces	United States	LMG 7866^T^

LMG: Laboratory of Microbiology Gent; ^T^: type strain; N.A.: not available.

**Table 2:** Isolation of spiked *E. albertii* from minced chicken and pork meat.

Meat	Type	Enrichment Time in h	Strain	Spiked Concentrations of 4 *E. albertii* Strains (CFU/g Meat)						
				10^6^	10^5^	10^4^	10^3^	10^2^	10^1^	10^0^
Chicken	minced	0	EH2338	4/4^a^	4/4	4/4	4/4	0/4	0/4	0/4
		0	14/1248	4/4	4/4	4/4	4/4	0/4	0/4	0/4
		0	NIAH_Bird_23	4/4	4/4	4/4	4/4	0/4	0/4	0/4
		0	LMG 20976^T^	4/4	4/4	4/4	4/4	0/4	0/4	0/4
		24	EH2338	4/4	4/4	4/4	4/4	4/4	4/4	4/4
		24	14/1248	4/4	4/4	4/4	4/4	4/4	4/4	4/4
		24	NIAH_Bird_23	4/4	4/4	4/4	4/4	4/4	4/4	4/4
		24	LMG 20976^T^	4/4	4/4	4/4	4/4	4/4	4/4	4/4
		48	EH2338	2/4	2/4	2/4	2/4	2/4	2/4	2/4
		48	14/1248	2/4	2/4	2/4	2/4	2/4	2/4	2/4
		48	NIAH_Bird_23	2/4	2/4	2/4	2/4	2/4	2/4	2/4
		48	LMG 20976^T^	2/4	2/4	2/4	2/4	2/4	2/4	2/4
Pork	minced	0	NIAH_Bird_23	1/1^b^	1/1	1/1	1/1	0/1	0/1	0/1
		24	NIAH_Bird_23	1/1	1/1	1/1	1/1	1/1	1/1	1/1
		48	NIAH_Bird_23	1/1	1/1	1/1	1/1	1/1	1/1	1/1

4/4^a^:1/1^b^: number of biological replicates having colourless colonies on TS-Albertii Agar, identified as *E. albertii* by MALDI-TOF MS, from total no. of biological replicates. ^T^: type strain.

**Table 3:** Detection of *E. albertii* by isolation, PCR, and plate washing.

Sample Type		Isolation			PCR			Plate Washing			
		Sample No.	No. of Positive Samples		Sample No.	No. of Positive Samples		Sample No.	No. of Positive Samples	Sample No.	No. of Positive Samples
			Pre- Enrichment	Post- Enrichment		24 h Enrichment	48 h Enrichment		Pre- Enrichment		Post- Enrichment
Beef	minced	51	0	0	35	0	0	10	0	19	0
	fillets	45	0	0	37	0	0	9	0	19	0
Pork	minced	30	0	0	19	0	0	10	0	9	0
	fillets	50	0	0	42	0	0	8	0	22	0
Chicken	minced	35	0	0	26	1 ^b^	1 ^b^	13	0	15	0
	fillets	51	1 ^a^	1	44	2 ^c^	2 ^c^	31	1 ^a^	31	0
	carcass	30	0	0	19	0	0	3	0	3	0
	total	292	1	1	222	3	3	84	1	118	0

^a^, ^b^, and ^c^ indicate the corresponding samples.

## Data Availability

The original contributions presented in the study are included in the article/supplementary material, further inquiries can be directed to the corresponding author.

## Supplementary Materials

The following supporting information can be downloaded at: https://www.mdpi.com/article/10.3390/microorganisms12122408/s1, Table S1: Determination of growth performance of four *E. albertii* strains after 24 h enrichment in TSB versus TSB-Albertii Broth, followed by plating on TSA versus TSA-Albertii Agar plates at 37° and 41.5 °C for 24 h aerobically; Table S2: PCR detection of *E. albertii* from spiked meat samples, 24 h post-enrichment.
