# Supplementary material for: Isolation and Detection of the Emerging Pathogen Escherichia albertii in Clinical Stool Samples and the Potential Transmission by Meat Samples in Retail
Source: Microorganisms. 2024 Nov 23;12(12):2408. doi: 10.3390/microorganisms12122408 (PMC11677093; doi:10.3390/microorganisms12122408)
Supplement: Supplementary file 1 [file microorganisms-12-02408-s001.zip › Supplementary Table S2.pdf]

Supplementary Table S2. PCR-detection of *E. albertii* from spiked meat samples, 24 h post-enrichment

Meat type		Strain	Spiked concentrations of *E. albertii* strains (CFU/g meat)						
			**10^6**	**10^5**	**10^4**	**10^3**	**10^2**	**10^1**	**10^0**
Chicken	carcass	NIAH_Bird_23	1/1[b]	1/1	1/1	1/1	1/1	1/1	1/1
	fillet	NIAH_Bird_23	1/1	1/1	1/1	1/1	1/1	1/1	1/1
	minced	NIAH_Bird_23	1/1	1/1	1/1	1/1	1/1	1/1	1/1
Beef	fillet	NIAH_Bird_23	1/1	1/1	1/1	1/1	1/1	1/1	1/1
	minced	NIAH_Bird_23	1/1	1/1	1/1	1/1	1/1	1/1	1/1
Pork	fillet	NIAH_Bird_23	1/1	1/1	1/1	1/1	1/1	1/1	1/1
	minced	NIAH_Bird_23	1/1	1/1	1/1	1/1	1/1	1/1	1/1

1/1[b]: Number of biological replicates yielding successful PCR amplification from the total number of biological replicates
